# The biomarkers of key miRNAs and gene targets associated with extranodal NK/T-cell lymphoma

**DOI:** 10.1515/med-2021-0409

**Published:** 2022-01-04

**Authors:** Yin-yin Peng, Hong-bin Zhang, Xin Wang, Qing Xiao, Shu-liang Guo

**Affiliations:** Department of Hematology Medicine, The First Affiliated Hospital of Chongqing Medical University, Chongqing, 400016, China; Department of Respiratory and Critical Care Medicine, The First Affiliated Hospital of Chongqing Medical University, Chongqing 400016, China

**Keywords:** extranodal NK/T-cell lymphoma, differentially expressed, microRNA, gene ontology, hub gene

## Abstract

Gene expression profiling studies have shown the pathogenetic role of oncogenic pathways in extranodal natural killer/T-cell lymphoma (ENKL). In this study, we aimed to identify the microRNAs (miRNAs) playing potential roles in ENKL, and to evaluate the genes and biological pathways associated to them. Gene expression profiles of ENKL patients were acquired from the gene expression omnibus (GEO) database. Most differentially expressed (DE)-miRNAs were identified in ENKL patients using limma package. Gene targets of the DE-miRNAs were collected from online databases (miRDB, miRWalk, miRDIP, and TargetScan), and used in Gene ontology (GO) and Kyoto encyclopedia of genes and genomes (KEGG) analyses on Database for annotation, visualization, and integrated discovery database, and then used in protein–protein interaction (PPI) analysis on STRING database. Hub genes of the PPI network were identified in cytoHubba, and were evaluated in Biological networks gene ontology. According to the series GSE31377 and GSE43958 from GEO database, four DE-miRNAs were screened out: hsa-miR-363-3p, hsa-miR-296-5p, hsa-miR-155-5p, and hsa-miR-221-3p. Totally 164 gene targets were collected from the online databases, and used in the GO and KEGG pathway analyses and PPI network analysis. Ten hub genes of the PPI network were identified: AURKA, TP53, CDK1, CDK2, CCNB1, PLK1, CUL1, ESR1, CDC20, and PIK3CA. Those hub genes, as well as their correlative pathways, may be of diagnostic or therapeutic potential for ENKL, but further clinical evidence is still expected.

## 1 Introduction

Natural killer (NK) cell tumors are categorized as extranodal NK/T-cell lymphoma (ENKL), aggressive NK-cell leukemia, chronic NK-cell lymphoproliferative disorders, etc., according to the 2016 World Health Organization (WHO) classification [1,2]. Among them, ENKL is relatively more common, which include both nasal and extra-nasal ENKL categories of disease in the current 2016 WHO classification [3]. ENKL is rarely diagnosed in Western countries, but relatively more common in East Asian countries [2]. ENKL is associated with Epstein–Barr virus (EBV) because EBV can infect T and NK cells and lead to EBV-associated T/NK cell lymphoproliferative disorders [1], according to observations of EBV molecules in tumor tissue and the correlations between EBV load and disease diagnosis and prognosis [4]. ENKL is a unique clinical entity characterized by an aggressive clinical course, and the prognosis is poor [5].

The pathogenesis of this tumor is poorly understood, but in recent years gene expression profiling studies have demonstrated the pathogenetic role of several oncogenic pathways in ENKL, such as AKT, STAT3, NF-KB, Notch-1, and Aurora kinase A [6,7]. In this sense, an important task is to find novel molecular and biological candidates for diagnostic, predictive, and prognostic potential in ENKL, including microRNAs (miRNAs).

miRNAs are small non-coding RNAs, which can regulate the gene expression in many cellular processes, such as cell proliferation, differentiation, and tumor genesis [8]. Since the discovery of miRNAs in circulation, a large amount of miRNA-related biological studies have confirmed that each miRNA can affect numerous target mRNAs [9] and each mRNA can be targeted by multiple miRNAs, enabling wide regulatory complexity of gene expression adjustment [10]. Furthermore, quantification of miRNAs can have potential diagnostic and prognostic utility in lymphoma [11,12,13]. miRNAs are easily detectable, relatively stable, and tissue specific, making them attractive candidate biomarkers [14]. Enhancing our understanding of the relationships between miRNAs and gene targets can help reveal detailed mechanisms and identify novel biomarkers for lymphoma.

In this study, we aimed to identify the miRNAs playing potential roles in the ENKL patients, which might further help provide valuable advice in clinical treatment. To that end, we acquired typical gene expression profiles of ENKL patients from the Gene Expression Omnibus (GEO) database, and identified four miRNAs that are most differentially expressed (DE) in those ENKL patients using limma package in R. After that, gene targets of these four DE-miRNAs were collected by employing online databases, and then in terms of those gene targets, Gene Ontology (GO) and Kyoto Encyclopedia of Genes and Genomes (KEGG) analyses were performed on the Database for Annotation, Visualization, and Integrated Discovery (DAVID), protein–protein interaction (PPI) were performed on STRING, and finally hub genes of the PPI network were identified in cytoHubba and evaluated in Biological Networks Gene Ontology (BiNGO) tool, in Cytoscape. Hope our study is helpful for revealing the mechanisms of ENKL pathogenesis, and for identifying the diagnostic and therapeutic biomarkers of ENKL.

## 2 Methods

### 2.1 Data source

The expression profiles of ENKL patients were collected from the GEO database. We searched the GEO for series about the expressions of miRNAs in ENKL patients which must contain both the tumor and normal samples, and only two series GSE31377 [15] and GSE43958 [16] were regarded suitable for this study. In GSE31377, we regarded the samples of ENKL patients’ tissues and NK tumor cell-lines as “ENKL” group, and those of normal NK cells from healthy individuals’ blood and ENKL patients’ normal tissues as “normal” group, while in GSE43958, we took the NK tumor cell-line samples as “ENKL” group, and those of normal NK cells from healthy individuals’ blood as “normal” group (Table 1). Differential expression analysis was performed between “ENKL” and “normal” groups in both series, separately.

**Table 1:** Sample grouping of GSE31377 and GSE43958 for DE-miRNA identification

Series	Groups	Sources of samples
GSE31377	ENKL	ENKL patients’ tissues and NK tumor cell-lines
	Normal	Normal NK cells from healthy individuals’ blood and ENKL patients’ normal tissues
GSE43958	ENKL	NK tumor cell-lines
	Normal	Normal NK cells from healthy individuals’ blood

### 2.2 Identification of DE-miRNAs

The limma R package v3.12 was applied and served as the processor. An R GUI application v1.77 with R v4.0.4 was installed via home-brew 3.0 with –*cask* option on Mac OS Catalina. The limma package was installed in the package installer tool of this application. DE-miRNAs of the normal and ENKL groups were screened out according to the criteria adjusted *p* < 0.05 and absolute log fold change |log_2_ FC| >1, where the FDR method was used to adjust the *p*, with an option *adjust=“BH”* for the toptable( ) function of limma. As both series GSE31377 and GSE43958 gave DE-miRNA lists, the common DE-miRNAs appearing in both lists were collected for subsequent analysis.

### 2.3 miRNA-target interactions

Investigating the gene targets of miRNAs is crucial for identifying the regulatory mechanisms and functions of miRNAs. Herein we predicted the gene target of the DE-miRNAs by employing four miRNA-target prediction tools: miRDIP, miRWalk, miRDB, and TargetScan. The gene targets were determined by overlapping results from the four websites: first, for each miRNA, gene targets from the four websites were overlapped and common ones were picked up as a candidate list; and second the candidate lists of the four miRNAs were merged. After the miRNA targets were determined, the interactions of them would be visualized in Cytoscape.

### 2.4 GO and KEGG pathway enrichment analyses of gene targets

GO annotation and KEGG pathway enrichment analyses of the determined gene targets were performed using the DAVID, which revealed gene enrichments associated with biological processes (BPs), cellular components (CCs), molecular functions (MFs), and KEGG pathways associated with the gene targets. Number of the gene targets enriched in each of those items would be listed.

### 2.5 PPI network and biological analysis of hub genes

To gain insights into the interactions between gene targets, a PPI network was constructed using the STRING tool to reveal the molecular mechanisms of ENKL. The STRING was able to give a complex full PPI network, and usually the interactions between the hub genes were more interesting. The full network from the STRING was imported into the cytoHubba plugin of the Cytoscape to identify the hub genes. For biological analysis of hub genes, another plugin of Cytoscape BiNGO was employed. BiNGO has the capability to evaluate and visualize the pathways of enriched BPs, CCs, and MFs. We only performed the analysis on enriched BPs of the ten hub genes in this study.

### 2.6 Statistical analysis

Statistical analysis was used only for the identification of DE-miRNAs. Specifically, when applying the limma package, first, a linear model *lmfit( )* would be fit for the expression data to estimate the expression levels of genes in each group, and then contrast fit *contrasts.fit( )* was performed to estimate the expression differences of genes between ENKL group and control group. After that, empirical Bayes statistics *ebayes( )* was employed for t-statistics, moderated F-statistics, and log-odds of differential expression, and finally top DE-miRNAs were exported via *toptable( )* of limma, where *p* values of the t-statistics were adjusted using FDR method with the option *adjust=“BH”*. The significant DE-miRNAs of each series that we were interested in were selected according to the criterion adjusted *p* < 0.05, and |log_2_ FC| > 1.


**Statement of ethics:** Ethical approval was not required in this study because all the data were collected from public papers and databases.

## 3 Results

### 3.1 DE-miRNAs: hsa-miR-363-3p, hsa-miR-296-5p, hsa-miR-155-5p, and hsa-miR-221-3p

**Figure 1:**
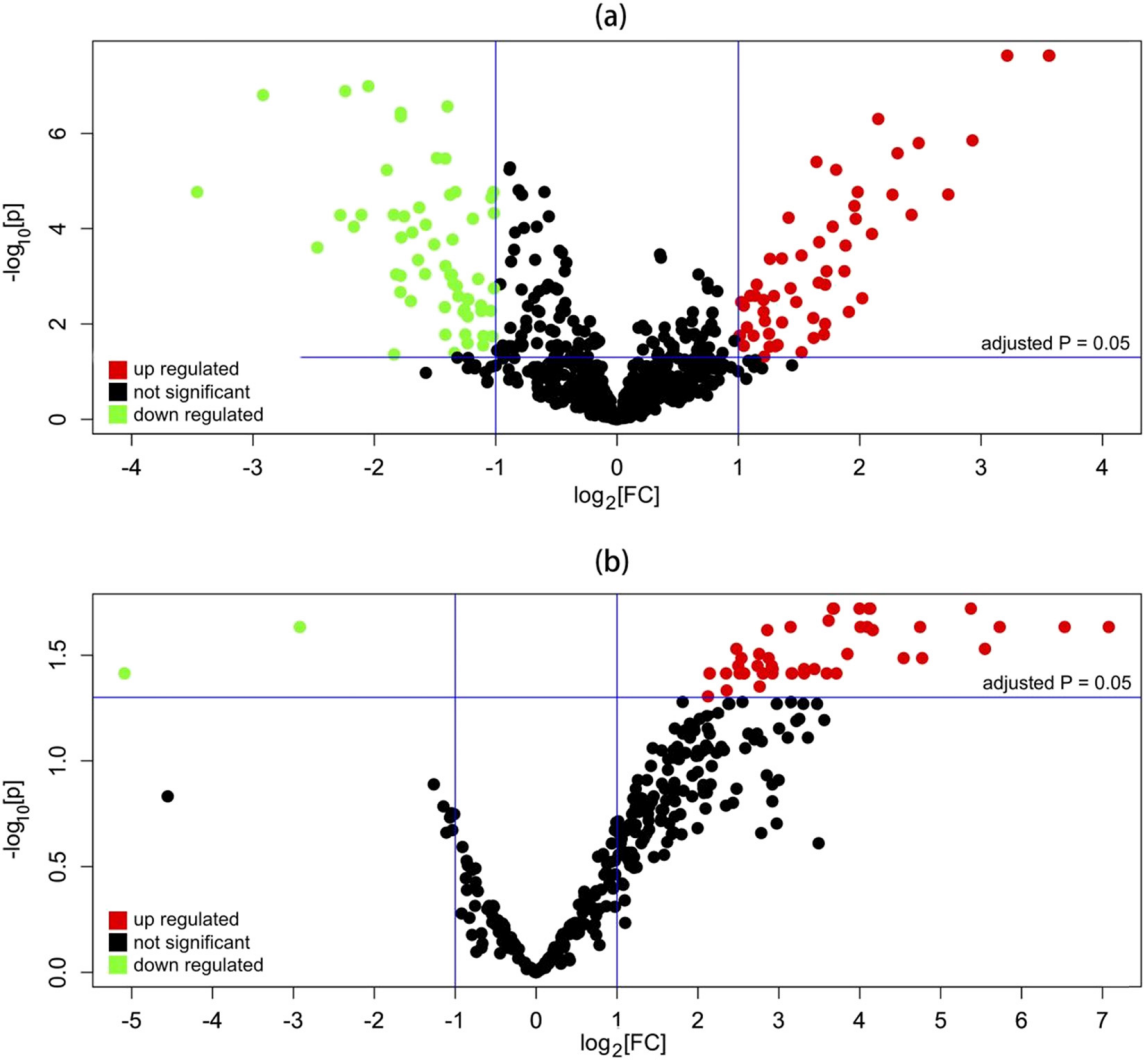
Volcano plots of the expressions of miRNAs for (a) GSE31377, and (b) GSE43958.

According to GSE31377, 229 DE-miRNAs (including 59 downregulated and 170 upregulated) were screened out, while for GSE43958, 49 DE-miRNAs (including 47 upregulated and 2 downregulated) were found (Figure 1 and Table 2). The common four appearing in both the DE-miRNA lists were finally selected for further analysis: hsa-miR-363-3p (down), hsa-miR-296-5p (up), hsa-miR-155-5p (up), and hsa-miR-221-3p (up in GSE43958 but down in GSE31377) (Table 3).

**Table 2:** Upregulated and downregulated miRNAs for series GSE31377 and GSE43958

GSE31377	Upregulated	hsa-miR-218-1*, hsa-miR-486-3p, hsa-miR-499-3p, hsa-miR-520a-5p, hsa-miR-633, hsa-miR-582-3p, hsa-miR-411*, hsa-miR-1233, hsa-miR-635, hsa-miR-541, hsa-miR-138-1*, hsa-miR-758, hsa-miR-637, hcmv-miR-UL148D, ebv-miR-BART20-5p, hsa-miR-1236, hsa-miR-196a*, hsa-miR-661, hsa-miR-501-3p, hsa-miR-625*, kshv-miR-K12-9*, hsa-miR-518b, hcmv-miR-UL112, ebv-miR-BART10*, hsa-miR-133a, hsa-miR-200a*, hsa-miR-486-5p, ebv-miR-BART13*, ebv-miR-BHRF1-1, hsa-miR-602, hsa-miR-596, ebv-miR-BART7*, hsa-miR-18b*, ebv-miR-BART20-3p, hsa-miR-583, hsa-miR-765, hsa-miR-493, hsa-miR-518c*, ebv-miR-BART2-5p, ebv-miR-BART9*, kshv-miR-K12-8, hsa-miR-566, hsa-miR-648, hsa-miR-483-5p, hsa-miR-1228, hsa-miR-639, hsa-miR-130b, hsa-miR-622, hsa-miR-155, hsa-miR-296-5p, hsa-miR-542-5p, kshv-miR-K12-3, hsa-miR-424*, ebv-miR-BART2-3p, ebv-miR-BART12, hsa-miR-630, hsa-miR-188-5p, hsa-miR-940, hsa-miR-345, hsa-miR-150*, ebv-miR-BART14*, kshv-miR-K12-10a, hsa-miR-572, hsa-miR-431, ebv-miR-BART18-3p, hsa-miR-135a*, ebv-miR-BART16, hsa-miR-575, hsv1-miR-LAT, ebv-miR-BART11-5p, hsa-miR-1224-5p, hcmv-miR-UL70-3p, hsa-miR-10b*, ebv-miR-BART11-3p, ebv-miR-BART13, ebv-miR-BART19-5p, hsa-miR-638, hsa-miR-614, hiv1-miR-H1, ebv-miR-BART19-3p, hsa-miR-801, ebv-miR-BART5, hsa-miR-663, ebv-miR-BART18-5p, hsa-miR-494, ebv-miR-BART4, ebv-miR-BART6-5p, ebv-miR-BART1-5p, hsa-miR-923, ebv-miR-BART8, ebv-miR-BART17-3p, ebv-miR-BART6-3p, ebv-miR-BART17-5p, ebv-miR-BART10, ebv-miR-BART8*, ebv-miR-BART1-3p, ebv-miR-BART3, ebv-miR-BART7, ebv-miR-BART9, and ebv-miR-BART14
	Downregulated	hsa-miR-150, hsa-miR-363, hsa-miR-26b, hsa-miR-101, hsa-miR-338-3p, hsa-miR-374a, hsa-miR-30b, hsa-miR-873, hsa-miR-28-5p, hsa-miR-140-5p, hsa-miR-142-5p, hsa-miR-768-3p, hsa-miR-29c, hsa-miR-30e, hsa-miR-152, hsa-miR-181a-2*, hsa-miR-374b, hsa-miR-186, hsa-miR-194, hsa-miR-340, hsa-miR-30c, hsa-miR-342-5p, hsa-miR-32, hsa-miR-361-3p, hsa-miR-148b, hsa-miR-10a, hsa-miR-22, hsa-miR-876-3p, hsa-miR-26a, hsa-miR-33a, hsa-miR-598, hsa-miR-876-5p, hsa-miR-148a, hsa-miR-140-3p, hsa-miR-590-5p, hsa-miR-30e*, hur_5, hsa-miR-744, hsa-miR-181c, hsa-miR-192, hsa-miR-22*, hsa-let-7c, hsa-miR-23a, hsa-miR-141, hsa-miR-27a, hsa-miR-98, hsa-miR-23b, hsa-miR-361-5p, hsa-miR-195, hsa-miR-215, hsa-miR-221, hsa-miR-185, hsa-miR-425, hsa-miR-151-5p, hsa-miR-95, hsa-miR-7-1*, hsa-miR-339-5p, hsa-miR-362-3p, hsa-miR-29c*, hsa-miR-200b, hsa-miR-132, hsa-miR-221*, hsa-miR-27b, hsa-let-7d, hsa-let-7b, hsa-miR-101*, hsa-miR-106a*, hsa-miR-138-2*, hsa-miR-191, hsa-miR-32*, hsa-miR-497, hsa-miR-505, hsa-miR-627, hsa-miR-122, hsa-miR-31, hsa-miR-30a, hsa-miR-423-5p, hsa-miR-660, hsa-miR-505*, hsa-miR-454, hsa-miR-539, hsa-miR-324-5p, hsa-miR-885-3p, hsa-miR-331-3p, hsa-miR-532-3p, hsa-miR-545, hsa-miR-30d, hsa-miR-625, hsa-let-7i*, hsa-miR-26b*, hcmv-miR-US25-2-5p, hsa-miR-196b, hsa-miR-190b, dmr_285, hsa-miR-132*, hsa-miR-29b-2*, hsa-miR-29a*, hsa-miR-513a-3p, hsa-miR-335, hsa-miR-340*, hsa-miR-769-5p, hsa-miR-499-5p, hsa-miR-595, hsa-miR-30d*, hsa-miR-624*, hsa-miR-339-3p, hiv1-miR-N367, hsa-miR-146b-3p, hsa-miR-197, hsa-miR-297, hsa-miR-374b*, hsa-miR-642, hsa-miR-326, hsa-let-7g*, hsa-miR-548c-5p, hsa-miR-148b*, hsa-miR-520f, hsa-miR-190, hsa-miR-449b, hsa-miR-186*, hsa-miR-188-3p, hsa-miR-185*, hsa-miR-590-3p, hsa-miR-593, hsa-miR-200c*, hsa-miR-24-1*, hsa-miR-519a, hsa-miR-374a*, hsa-miR-577, hsa-miR-592, hsa-miR-578, hsa-miR-363*, and hsa-miR-573
GSE43958	Upregulated	hsa-miR-20a-5p, hsa-miR-17-5p, hsa-miR-19b-3p, hsa-miR-106a-5p, hsa-miR-20b-5p, hsa-miR-1275, hsa-miR-1973, hsa-miR-1246, hsa-miR-19a-3p, hsa-miR-155-5p, hsa-miR-29b-3p, hsa-miR-210-3p, hsa-miR-3648, hsa-miR-3197, hsa-miR-4284, hsa-miR-711, hsa-miR-4294, hsa-miR-3154, hsa-miR-221-3p, hsa-miR-3679-5p, hsa-miR-29a-3p, hsa-miR-642b-3p, hsa-miR-3687, hsa-miR-3175, hsa-miR-370-3p, hsa-miR-1281, hsa-miR-1825, hsa-miR-3141, hsa-miR-4279, hsa-miR-3651, hsa-miR-423-5p, hsa-miR-92a-3p, hsa-miR-1908-5p, hsa-miR-1976, hsa-miR-4299, hsa-miR-1913, hsa-miR-301a-3p, hsa-miR-1274a, hsa-let-7f-5p, hsa-miR-21-5p, hsa-miR-296-5p, hsa-miR-92b-3p, hsa-miR-320a-3p, hsa-miR-4327, hsa-miR-1236-3p, hsa-miR-1229-3p, and hsa-miR-4323
	Downregulated	hsa-miR-363-3p and hsa-miR-150-5p

**Table 3:** The four common DE-miRNAs of GSE31377 and GSE43958 series

	GSE31377		GSE43958	
	log_2_ FC	Adjusted *p*	log_2_ FC	Adjusted *p*
hsa-miR-363-3p	−2.92	1.57 × 10^−7^	−2.92	0.023
hsa-miR-155-5p	1.04	0.004	7.08	0.023
hsa-miR-221-3p	−1.12	0.005	2.76	0.031
hsa-miR-296-5p	1.07	0.012	2.80	0.039

### 3.2 164 gene targets and interactions were identified for DE-miRNAs

After searching the four online databases, finally 164 genes associated with the four DE-miRNAs were determined as the ones playing potential roles in ENKL pathogenesis (Table 4). The bridge genes between hsa-miR-363-3p and hsa-miR-221-3p are FNIP2 and ANKIB1, and the other bridge gene between hsa-miR-221-3p and hsa-miR-155-5p is ZNF652, while the genes associated with hsa-miR-296-5p appear isolated from others.

**Table 4:** miRNA-target interactions of hsa-miR-363-3p, hsa-miR-296-5p, hsa-miR-221-3p, and hsa-miR-155-5p according to miRDIP, miRWalk, miRDB, and TargetScan

miRNA	Gene targets
hsa-miR-363-3p	ZDHHC5, ZDHHC3, XRN1, USP28, TTC28, TRAF3, TNRC6B, TMEM50B, TEF, SYT1, SYNJ1, SYNDIG1, SOX11, SLC25A36, SETD5, SEL1L3, RRN3, RGS3, RBPMS2, RASSF3, RAP1B, RAB23, PRKCE, POLK, PITPNM2, PIP4K2C, PIK3R3, PHLPP2, PAPOLA, OTUD3, NUFIP2, NSMAF, NFIX, NF2, MYO5A, MMP16, MBNL3, MAN2A1, LUZP1, LPP, LHFPL2, KIF5B, KCNC4, KAT2B, ITGAV, HOXD10, HAS3, GPR180, GPR158, FNIP2, FNIP1, FNDC3B, FAR1, FAM160B1, FAM133B, FAM126B, ELOVL6, DYRK2, DUS2, DCP1A, DACT1, DAB2IP, CPEB2, CD69, C21ORF91, C20ORF194, AURKA, APPL1, ANKRD44, ANKIB1, and ADAM19
hsa-miR-296-5p	TMEM135, TEAD3, NUAK2, NSD1, MACF1, KCTD15, GPC2, GAB2, FGFR1, FAM53B, EPN1, ECHDC1, DYNLL2, CMTM4, and BAHD1
hsa-miR-221-3p	ZNF652, UBN2, TNRC6C, TFG, TCF12, SYT10, SLC4A4, SLC25A37, SEMA6D, SBK1, PTBP3, POGZ, PLEKHA2, PCDHAC1, PCDHA3, PANK3, NRG1, NIPAL4, NFATC3, NDST3, NDFIP1, MIER3, MIDN, KPNA1, KMT2A, KLF7, KDR, KCNK2, IGF2BP2, HIPK1, GBX2, GABRA1, FRY, FNIP2, FAM214A, ETV3, ESR1, ERBB4, EML6, DYRK1A, DPP8, DLG2, DCUN1D4, DCUN1D1, DCAF7, CRKL, CPNE8, CDON, CACNB4, C6ORF120, C3ORF70, BRWD1, BMF, BEND4, BCL2L11, ATXN1, ATAD2B, ARID1A, ARHGEF7, ANKIB1, ANGPTL2, AKAP5, and ADAM22
hsa-miR-155-5p	ZNF652, XKR4, VAV3, TSPAN14, TRIM32, TM9SF3, SOX10, MYLK, KDM5B, KANSL1, HNRNPA3, FBXO11, DUSP14, CKAP5, C6ORF89, BNC2, ATXN1L, and AAK1

### 3.3 Enrichment of 164 gene targets in BPs, CCs, MFs, and KEGG pathways

**Figure 2:**
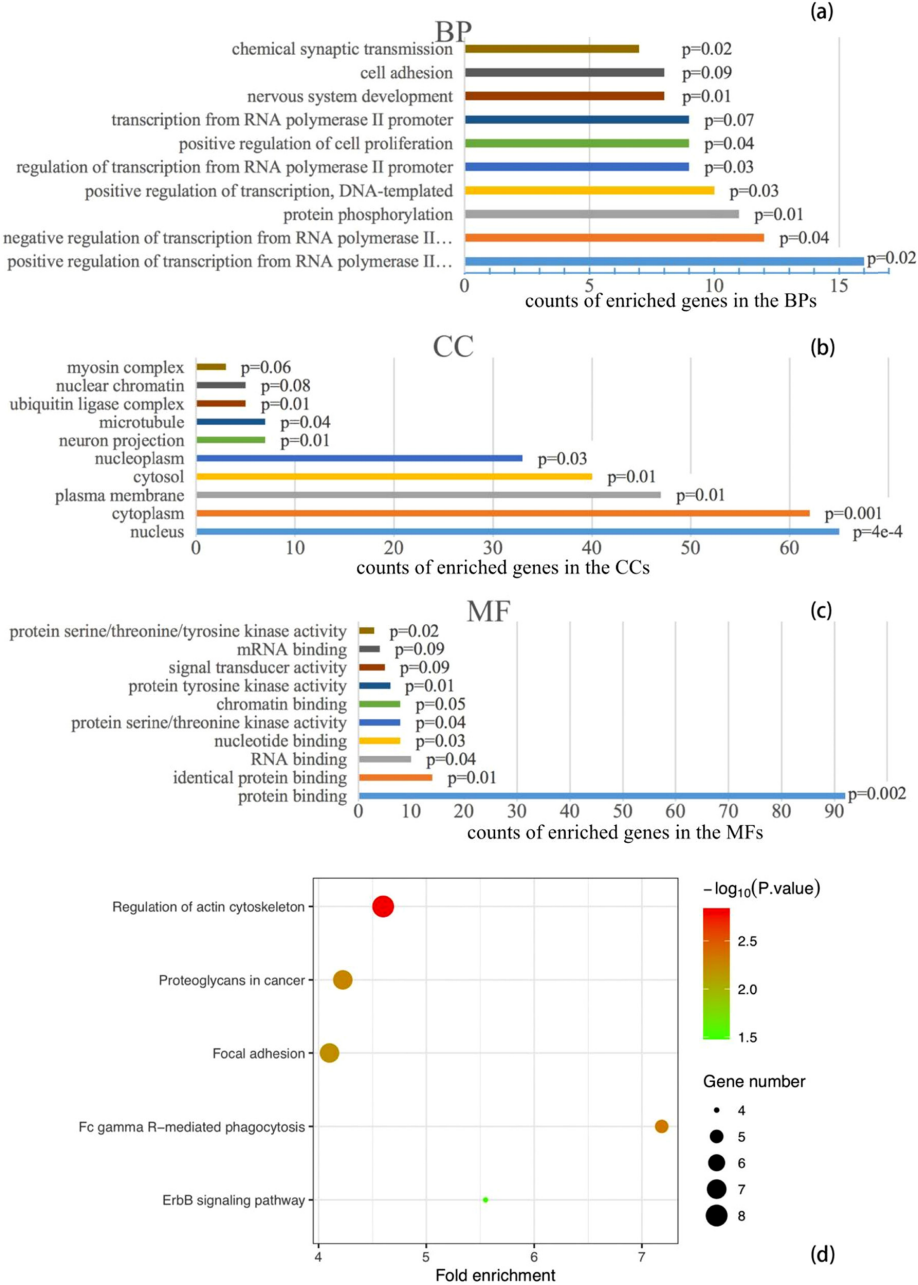
Top 10 gene counts of enrichment in BPs (a), CCs (b), and MFs (c), and KEGG pathways (d). Note: bar colors in (a), (b), and (c) only represent intuitive distinguishing of entries, not any scale of values.

Using the 164 gene targets, the DAVID listed the top 10 enriched gene counts of 61 BPs, 15 CCs, 18 MFs, and 10 KEGG pathways (Figure 2). The significantly enriched entries of BPs include positive and negative regulations of transcription from RNA polymerase II, and protein phosphorylation; the majority of the enriched CCs include nucleus, cytoplasm, plasma membrane, cytosol, and nucleoplasm; and the most enriched MFs is protein binding. The enriched entries of KEGG pathways include regulation of actin cytoskeleton, FcγR-mediated phagocytosis, proteoglycans in cancer, focal adhesion, ErbB signaling pathway, thyroid hormone signaling pathway, sphingolipid signaling pathway, microRNAs in cancer, rap1 signaling pathway, and pathways in cancer.

### 3.4 Hub genes of 164 gene targets: AURKA, TP53, CDK1, CDK2, CCNB1, PLK1, CUL1, ESR1, CDC20, and PIK3CA

**Figure 3:**
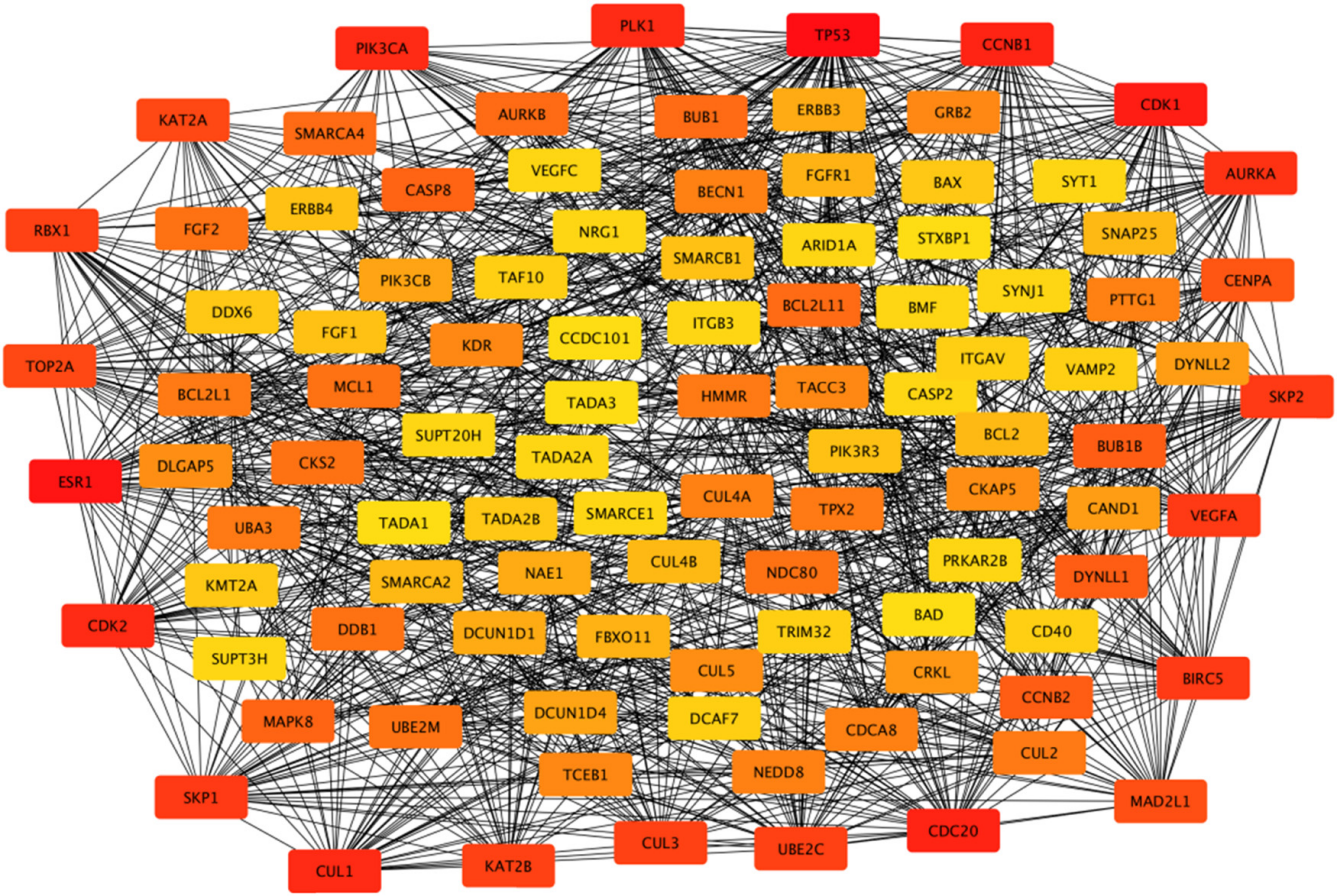
The PPI network associated with the 164 gene targets. Note: Box colors only represent relative comparison of connection counts of boxes in the network. Deeper red represents more connections.

**Figure 4:**
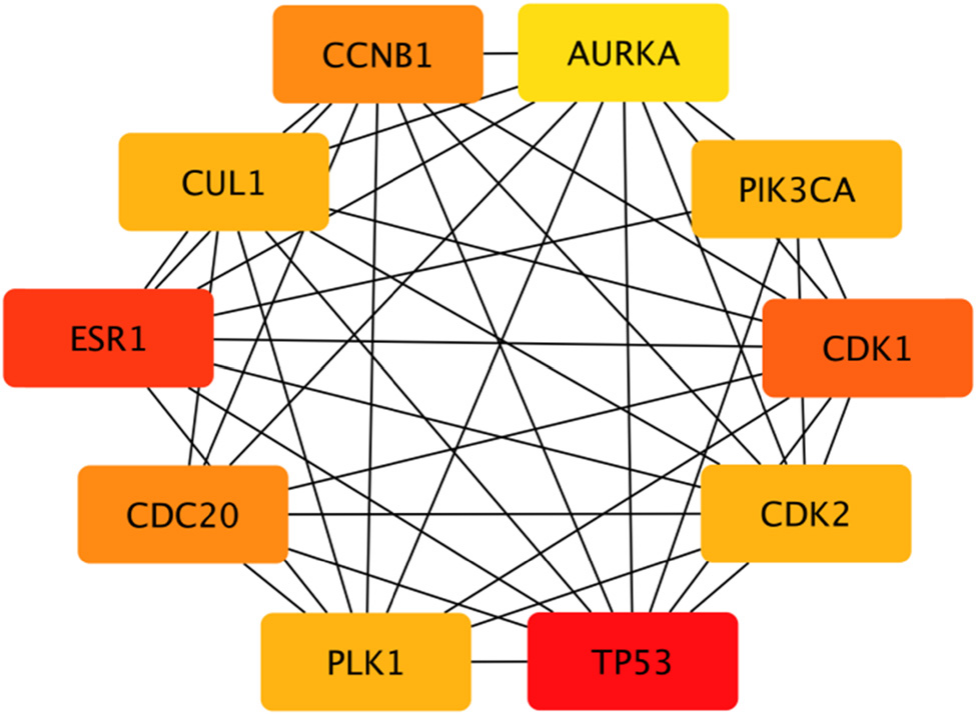
Top hub genes and their interactions of the PPI network. Note: Box colors only represent relative comparison of connection counts of boxes in Figure 3. Deeper red represents more connections.

**Figure 5:**
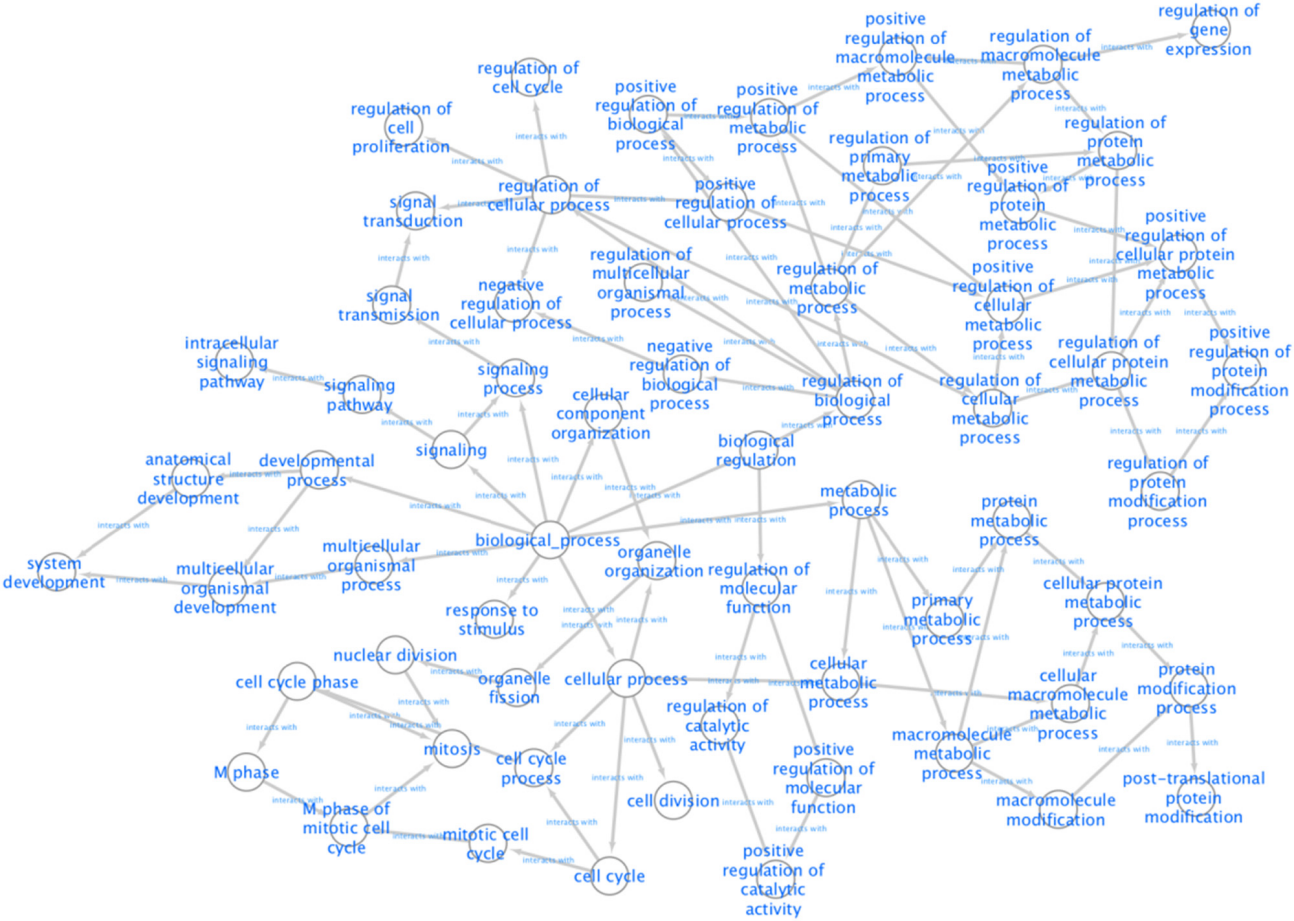
Pathways of enriched BPs according to results of the BiNGO.

According to the 164 gene targets, STRING gave a complex network with 1,638 lines and 253 nodes. A reduced graph of this network is shown in Figure 3, in which only the top 100 scored nodes are kept, and the node color represents the connection degree of node, evaluated in cytoHubba. Hub genes, playing essential potential roles in the network, were distinguished in terms of the connection degree. We selected the top 10 hub genes for further analysis: AURKA, TP53, CDK1, CDK2, CCNB1, PLK1, CUL1, ESR1, CDC20, and PIK3CA (Figure 4). Figure 5 is the biological pathways of enriched BPs of hub genes, where the nodes with associated gene count less than five have been removed for a more clear network. This biological process pathway network involves 64 nodes and 91 edges.

## 4 Discussion

ENKL is a highly aggressive tumor, and a better understanding about the molecular abnormalities could provide important insights into the biology of this disease, as well as potential new therapeutic choice. Numerous trials have indicated that miRNAs may play key roles in the development of ENKL. Accordingly, strategies for the diagnosis and treatment of ENKL might be furnished via analysis of correlative data in GEO database and construction of ENKL-associated miRNA-target interactions regulatory network.

In this study, we screened out four common DE-miRNAs from GEO series GSE31377 and GSE43958. Among the four DE-miRNAs, miR-155 has been reported in several hematologic malignancies, including acute myeloid leukemia [17,18], acute and chronic lymphoblastic leukemia [19,20], myelodysplastic syndrome [21], diffuse large B-cell lymphoma (DLBCL) [22], follicular lymphoma [22], Hodgkin’s lymphoma [23], and ENKL [24].

miRNA-155 is highly expressed in ENKL and NK tumor cell-lines where it activates the AKT signaling pathway to down-regulate tumor suppressor genes, leading to tumor cell proliferation [25]. As ENKLs are closely associated with EBV, miR-155 plays an important role in the regulating immune responses and the development of viral infections, indicating that this miRNA can promote the development of tumors by regulating the expression of inflammatory factors and changing the microenvironment [24,26,27]. Furthermore, inhibition of miR-155 expression could improve the sensitivity of ENKL to doxorubicin, even reverse the drug resistance [24,28]. Therefore, miR-155 is a key factor in the emergence and development of ENKL, making it a potential clinical indicator to evaluate the diagnosis, treatment, and prognosis of patients with ENKL.

Likewise, as for miR-363, several studies have revealed the possible regulatory functions of miR-363 in diverse BPs. The miR-363 was found downregulated in the Burkitt’s lymphoma than in normal tissue [29], and the downregulation of miR-363 could precisely differentiate the high-grade lymphomas into ABC or GCB subtypes [30]. Evidence has shown that the expression of miR-363 is correlated with clinical outcomes [30]. Moreover, the down-regulation of miR-363 has been shown to be associated with drug-resistance [31] and poor prognosis [32]. Its downregulation has also been detected in many solid tumors such as ovarian cancer, and in human papillomavirus-transfected keratinocyte HaCaT cells [33]. In this study, as the downregulation of miR-363-3p was only shown in bioinformatics, more information is needed for larger scale clinical verification.

The miR-221-3p in this study was found upregulated in GSE43958 but downregulated in GSE31377. In fact such difference has been noticed in clinical practice by Guo et al. [34]. Plasma miR-221 of 79 ENKL patients were measured in that study, and were found upregulated or downregulated differently. The authors compared the upregulated and downregulated groups, and found significant differences with respect to gender (*p* = 0.003) and ECOG performance status (*p* = 0.023), but insignificant with respect to their response rate and complete remission rates. They also found the upregulation of miR-221 associated with shorter overall survival. So it is indicated that miR-221 may either be upregulated or downregulated in ENKL patients, but upregulated miR-221 may be associated with poorer prognosis. We must note, however, that as the discrepancy of regulations of hsa-miR-221-3p exists in GSE43958 and GSE31377, the role of hsa-miR-221-3p in ENKL needs still further investigation.

miR-296 is located in chromosomal region 20q13.32. It has a high degree of sequence conservation among species and plays important roles in many BPs [35]. miR-296 is upregulated in various types of cancers, such as DLBCL, gastric cancer, etc. [36]. It functions as an oncogene by targeting important tumor suppressors, such as CDX1, STAT5A, SOCS2, ICAM1, CASP8, NGFR, NF2, and HGS, and thereby regulates important cancer-related processes, such as cell proliferation, apoptosis, invasion, migration, blood vessel development, and chemoresistance [36]. In this study, bioinformatics show that miRNA-296-5p was upregulated, and the TMEM135, TEAD3, NUAK2, NSD1, MACF1, KCTD15, GPC2, GAB2, FGFR1, FAM53B, EPN1, ECHDC1, DYNLL2, CMTM4, and BAHD1 are potential target genes of miRNA-296-5p, but no definitive evidence has yet been reported in literature. Our findings indicate that miR-296-5p might play roles in inflammatory and malignant tumors, but further clinical evidence is still expected.

Thus, more detailed research is needed to explore the potential functions of the identified DE-miRNAs in ENKL. We found that several hub genes, such as AURKA, TP53, CDK1, CDK2, CCNB1, PLK1, CUL1, ESR1, CDC20, and PIK3CA, and correlative pathways, including regulation of actin cytoskeleton, FcγR-mediated phagocytosis, proteoglycans in cancer, focal adhesion, and ErbB signaling pathway, may be of diagnostic or therapeutic potential for ENKL.

A wide range of diseases, including cancer, result from malfunctioning of actin cytoskeletal proteins [37] and proteoglycans [38]. So further studies and more clinical samples are underway to determine whether an actin cytoskeleton or proteoglycan panel targeted antibody could be useful in the classification and better characterization of ENKL. Focal adhesion kinase (FAK) is a promising target for the treatment of solid tumors because its expression has been linked to tumor progression, invasion, and drug resistance [39]. Several FAK inhibitors have been developed and tested for efficacy in treating advanced cancers [39]. As lymphoma’s biological behavior is more inclined to solid tumor [40], and we found focal adhesion to play an important role in ENKL, so FAK may serve as an effective therapeutic target to ENKL. Two important types of ErbB inhibitor are in clinical use: humanized antibodies directed against the extracellular domain of EGFR or ErbB2, and small-molecule tyrosine-kinase inhibitors that compete with ATP in the tyrosine-kinase domain of the receptor [41]. As we lack experience of ErbB inhibitor used for ENKL, so a larger scale verification for it is still needed. Hope in the future, further factors that underlie clinical response to signaling pathway targeted therapeutics could be uncovered by continuing the combination of fundamental and clinical studies. We also hope that the network of biological process pathways will contribute not only to the development of novel therapeutics, but also in allowing us to optimally use those already in the clinic.

## 5 Limitations

There are several limitations to our present study. (1) The number of samples we obtained from GSE31377 and GSE43958 is relatively small, and the data from the two series were not comprehensive enough, especially the amount of data in GSE43958 were very limited, even with plenty of blanks, which partially limits the result of this study. However, other series in GEO either contain fewer samples, or lack healthy samples as control group; (2) in GSE31377, the “ENKL” group contains samples of both ENKL tissue and NK tumor cell-lines, while in GSE43958, the “ENKL” group contains samples of only NK tumor cell-lines, thus uncertainties might be introduced because the miRNA and genes in NK tumor cell-lines may be different from those in ENKL tissues; (3) the normal NK cells were collected from healthy individuals’ blood, while ENKL tissue samples were from tissues. This difference in their sources may also affect the results. Thus, more samples are needed for validation with quantitative reverse transcription polymerase chain reaction in further research. In addition, the functions and molecular mechanisms of genes are very complicated, thus predictions based only on bioinformatics need cellular and animal experiments for verification.

## 6 Conclusion

In conclusion, by employing the GEO series GSE31377 and GSE43958 and bioinformatic analysis, we not only found four DE-miRNAs in ENKL: hsa-miR-363-3p, hsa-miR-155-5p, hsa-miR-221-3p, and hsa-miR-296-5p but also determined ten hub genes that may serve as potential biomarkers of ENKL: AURKA, TP53, CDK1, CDK2, CCNB1, PLK1, CUL1, ESR1, CDC20, and PIK3CA. Our findings might help improve the understanding of the pathogenesis of ENKL, help provide reliable biomarkers for precise diagnosis and individualized treatment of ENKL, and help discover novel therapeutics of ENKL in the future.

## Abbreviations

BiNGO: Biological networks gene ontology
BP: biological processes
CC: cellular components
DAVID: database for annotation, visualization, and integrated discovery
DE: differentially expressed
DLBCL: diffuse large B-cell lymphoma
EBV: Epstein–Barr virus
ENKL: extranodal NK/T-cell lymphoma
FAK: focal adhesion kinase
GEO: gene expression omnibus
GO: gene ontology
KEGG: Kyoto encyclopedia of genes and genomes
MF: molecular functions
miRNA: microRNA
PPI: protein–protein interaction
WHO: World Health Organization
